# Waste packaging polymeric foam for oil-water separation: An environmental remediation

**DOI:** 10.1016/j.dib.2018.05.033

**Published:** 2018-05-18

**Authors:** Chandrashekhar S. Patil, Vaibhav R. Patil, Sanket N. Anbhule, Chandrakant J. Khilare, Govind B. Kolekar, Anil H. Gore

**Affiliations:** a Department of Chemistry, Rajashri Chhatrapati Shahu College, Kolhapur 416005, Maharashtra, India; b Fluorescence Spectroscopy Research Laboratory, Department of Chemistry, Shivaji University, Kolhapur 416004, Maharashtra, India; c School of Nanoscience & Technology, Shivaji University, Kolhapur 416004, Maharashtra, India

**Keywords:** Waste, Packaging foam, Oil-water separation, Removal, Wastewater, Environmental remediation

## Abstract

Nowadays, its urgent need to develop and fabricate efficient, low cost, eco-friendly, oil-water separation methodologies especially for variety of polluted water in the environments. To deals with serious oil spills and industrial organic pollutants, here in we have developed a highly efficient oil-water separation methodology by using waste material such as expanded polyethylene (EPE) polymeric foam which is most commonly used for packaging as a shock absorber and most abundantly available in the surroundings as waste. Oil-water separation setup was fabricated by using waste EPE polymeric foam without any pre-treatment. By simply scratching, special properties (wettability performance) such as hydrophobicity, leophilicity, and low water adhesion was imparted to the EPE polymeric foam. The different types of oil-water mixture used for the study and separation were achieved almost up to 78%. The oil absorption efficiency of the EPE polymeric foam was within range of 0.491–0.788 g/g. In addition to efficient oil-water separation, the modified EPE polymeric foam exhibited fast and continuous oil-water separation solely by gravity. The easy operation, chemical durability, and efficiency of the waste EPE polymeric foam give it high potential for use in industrial and consumer applications for large scale oil-water separation.

**Specifications Table**

**Table:** 

Subject area	Chemistry/Environmental Pollution/Chemical engineering
More specific subject area	Oil-water separation, Wastewater treatment, Absorption, Surface Chemistry, Environmental Engineering
Type of data	Figures, Images, and Table
How data was acquired	All the experiments were performed as per the designed and optimized methodology. The separation efficiency (absorption capacity) and percentage of oil absorbed by the waste polymeric EPE foam was calculated as per the formula. The contact angle measurement carried out by using contact angle meter.
Data format	Processed and analyzed
Experimental factors	Waste packaging (EPE) polymeric foam was collected from the surrounding in the campus.
Experimental features	Waste packaging polymeric foam was used for oil-water separation.
Data source location	Kolhapur, Maharashtra, India
Data accessibility	Data are accessible with the article

**Value of the data**

• For purification of water, waste polymeric sponges are used.
• Devolved method used for separation of oil from water is economical one.
• It can be used for large scale separation oil-water from polluted (waste) water.
• To reduce the effect of oils on aquatic community and environment.
• Waste for waste for making best in environmental protection.

## 1 Data

Plastic products opened modern era in industrial history ever, since synthetic polymers were first introduced into industrial scale production in 1940. Plastic products are extensively used in terms of shopping bags, bottles, and pipes etc. As per as awareness of white pollution concerned presently various methods are used to degrade plastics via landfill, incineration, recycling and energy production [1]. Consequently, these processes resulting in the emission of toxic gases, fly ash, process waste etc. So, overwhelming use of plastic poses a threat to human being due to their non-degradable nature. Thus, there is an urgent need to extend technology which will utilize and recycle plastic wastes carefully.

In recent year’s marine pollutions due to oil spill events has occurred frequently which has attracted worldwide attention to addressing water pollution. These accidents causes’ significant economic loss and are like to have catastrophic effect on ecosystem. Especially the effluent coming out from industrial area and automobile washing centers are highly contaminated with oil as a hazardous pollutant. The frequency of water pollution caused by industrial oily waste and oil spills in ocean has increased tremendously in nowadays. Hence, to minimize water pollution, oil-water separation becomes an urgent need and challenge to the world-wide.

In this decade, efforts have been taken by the researchers for the development methodology for effective separation/removal of oil from polluted water [2], [3], [4], [5]. Recently, J. Saleem et al. and coauthors published review on oil sorbents from plastic wastes and polymers [6]. The reported methods in the review developed by the various researchers suffers its own limitations such as use of new polymers by purchasing from market so methods are not economical one, need to dissolve used polymer in organic solvent, lack of gravity/continuous separation (need to use vacuum pump), etc. and critically cannot used on large scale. Taking into account of all the above limitations, we inspired to develop methodology for oil-water separation based on waste based materials. Herein, we have used waste packaging EPE polymeric foam for efficient oil-water separation for from different type’s oils viz. cooking oil (used and unused), engine oil (used and unused), hair oil, diesel oil.

**Fig. 1:**
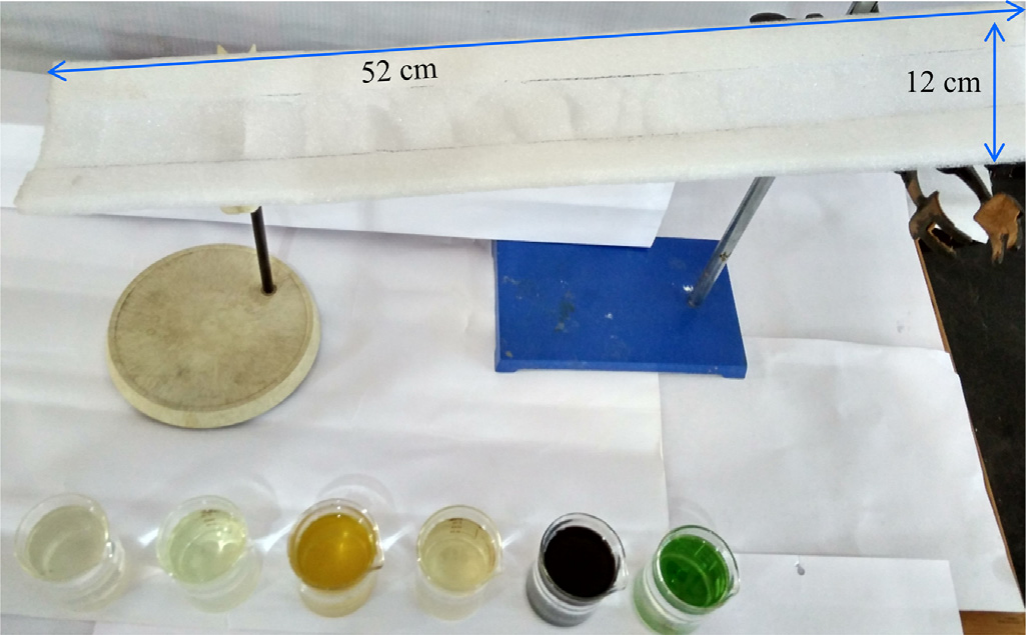
Showing scrubbed and pricked collected EPE polymeric foam fixed on stand with proper slope and angle for oil-water separation.

**Fig. 2:**
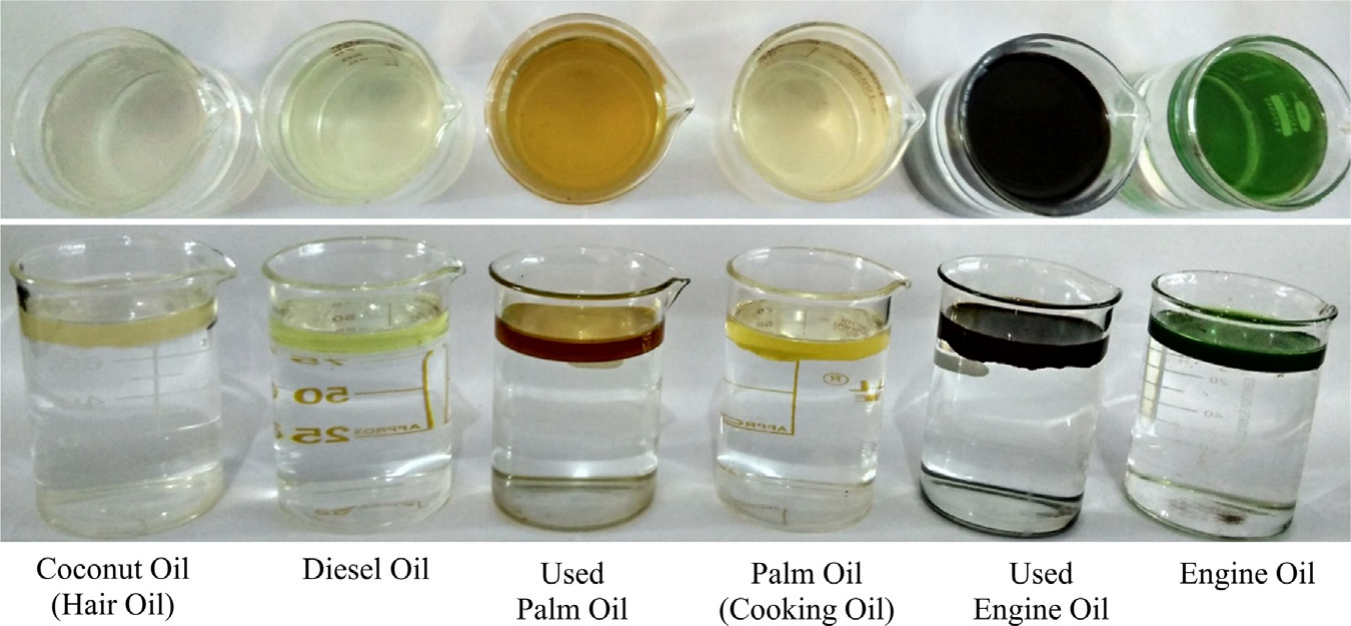
Prepared mixtures for different types of oil-water mixture (10:90 V/V) before separation.

**Fig. 3:**
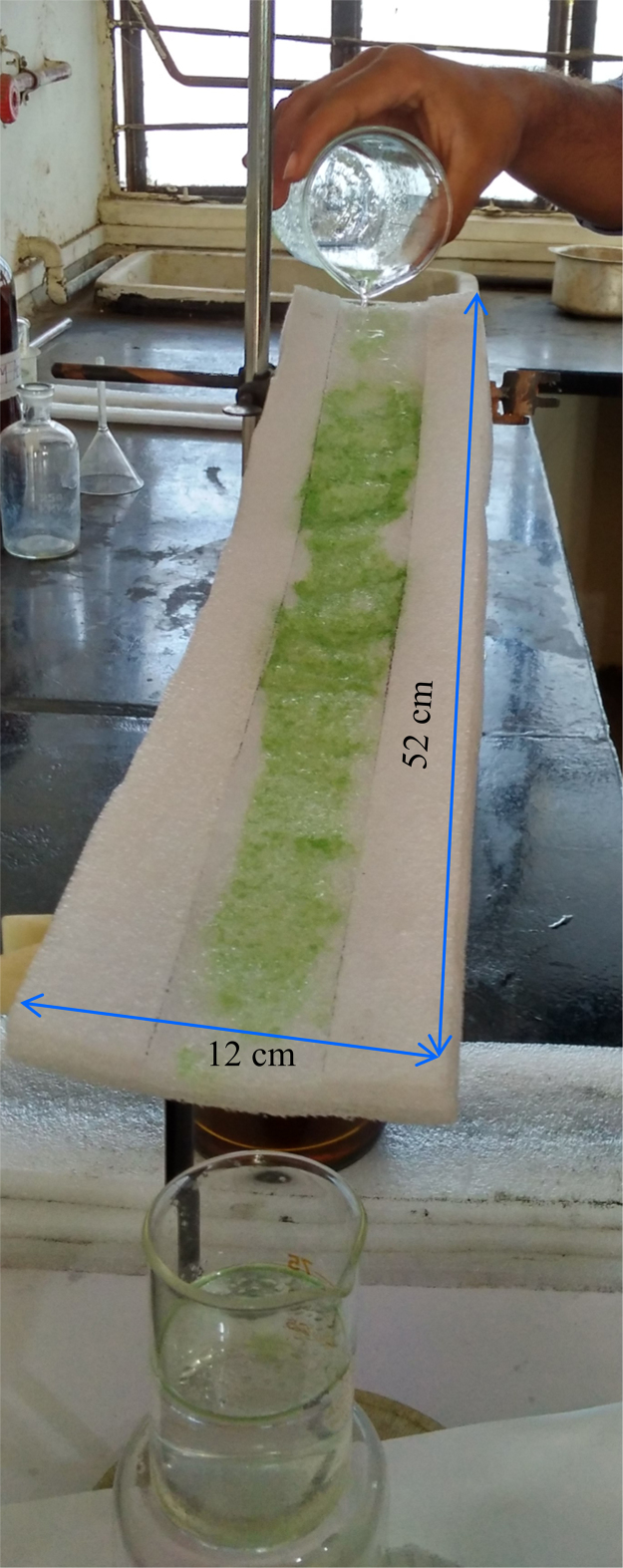
Showing actual experimental procedure during oil-water separation for engine Oil.

**Fig. 4:**
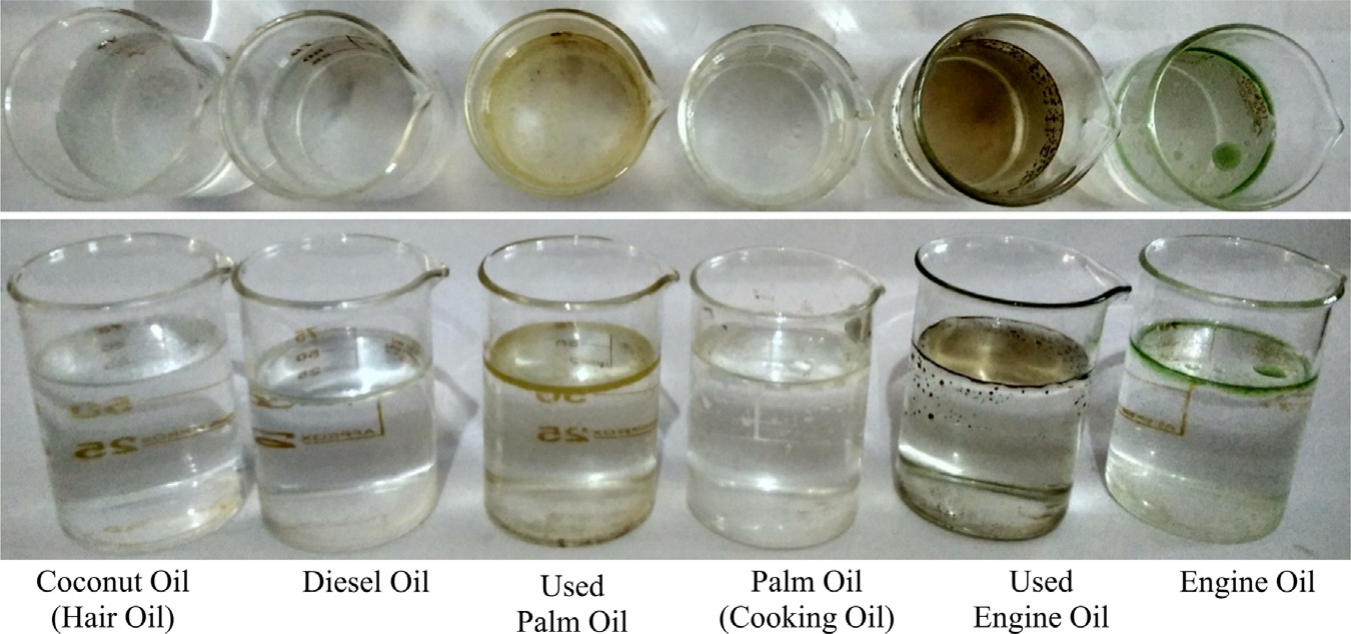
Showing photograph of different type’s oil-water mixture after separation.

**Fig. 5:**
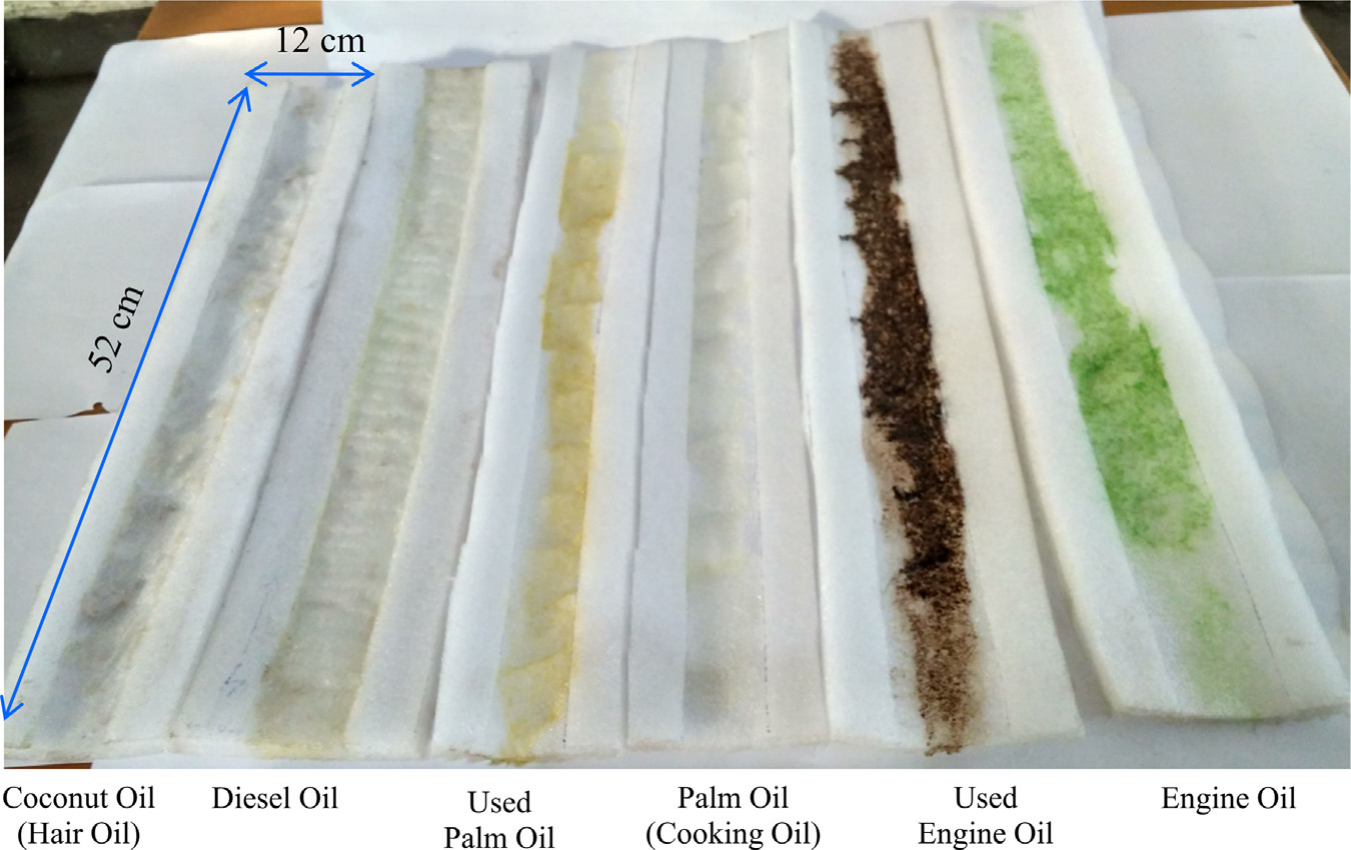
Showing photograph of different type’s oil absorbed on EPE polymeric foam after separation.

**Fig. 6:**
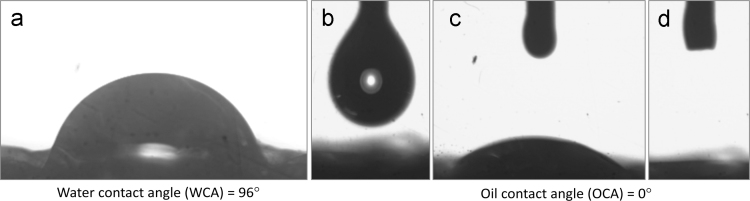
Images of contact angle measurement at different time intervals; (a) water droplet after 5 min, (b) engine oil droplet just before falling, (c) immediately after falling and (d) 5 sec. after falling on EPE foam.

There are in all total six figures and one table in this data article. Data in this article reflects the how we can use waste packaging polymeric EPE foam for the effective separation of oil from different types of oil-water mixtures. Fig. 1, Fig. 2, Fig. 3, Fig. 4, Fig. 5 shows details of experimental procedure used during the separation of oil-water mixture. The images of contact angle measurement for oil and water are shown in Fig. 6. The calculated oil absorption capacity (efficiency) and percentage of oil absorption for six different types of oil is systematically presented in Table 1.

**Table 1:** Results of oil-water separation by using waste polymeric EPE foam.

Sr. no	Type of oil	Volume of oil (mL )	Volume of water (mL)	Oil-Water mixture ratio (V:V)	Oil absorption efficiency Q (g/g or wt/wt )	Oil absorption percentage (%)
1.	Engine Oil	10	90	10:90	0.6656	66.56
2.	Palm Oil	10	90	10:90	0.7881	78.81
3.	Used engine Oil	10	90	10:90	0.6121	61.21
4.	Used palm Oil	10	90	10:90	0.5805	58.05
5.	Hair Oil	10	90	10:90	0.6273	62.73
6.	Diesel Oil	10	90	10:90	0.4911	49.11

From above table it was observed that maximum absorption capacity of EPE foam is towards Palm and minimum to the diesel oil. Moreover, EPE foam also shows absorption efficiency up to 78%.

## 2 Experimental design, materials and methods

### 2.1 Materials

Waste packaging EPE polymeric foam collected from dumping area near the campus. All types of oil samples such as Engine Oil, Palm Oil, Hair Oil and Diesel Oil were purchased from local market. Used Engine Oil and Used Palm Oil collected from automobile centre and from hotel respectively nearby in the campus.

### 2.2 Experimental procedure

First of all, we had collected waste packaging (EPE) polymeric foam from the surrounding in environment. Collected foam was washed with water to remove dirt and cut into specific shape and further scrubbed and pricked into specific 12 cm × 52 cm size. After that, scrubbed foam sheet was fixed on stand in laboratory with proper slope and angle. Then we had prepared 10: 90 (oil: water) mixtures for different types of oil. Furthermore, these mixtures were fed at top end of scrubbed EPE with proper slope and then water was rejected and oil was absorbed by the EPE sheets and this separated water was collected in the beaker at the lower end of EPE polymeric foam. The oil was collected by squeezing the foam (Fig. 1, Fig. 2, Fig. 3, Fig. 4, Fig. 5, Fig. 6). The contact measurement of water and engine oil sample was carried out by using contact angle meter (Holmarc, HO-IAD-CAM-01B) equipment with CCD camera.

### 2.3 Calculation of absorption capacity

The absorption capacity and percentage of oil absorbed by waste EPE foam is calculated by using following Eq. (1) and Eq. (2) respectively.

(1) $$Q=\frac{m_{A}\;-\;m_{0}}{m_{0}}$$

(2) $$\mathrm{Percentage of Absorption}(\%)=\frac{m_{A}\;-\;m_{0}}{m_{0}}\;\times 100$$

Where, m_A_ = Weight of foam in gram after absorption, m_0_ = Weight of foam in gram before absorption and Q = Absorption capacity (g/g or wt/wt)

### 2.4 Contact angle measurement

Fig. 6 shows images of contact measurement for water and oil droplets on EPE foam. The calculated values of contact angle measurement are as follows;

(i) Water contact angle (WCA) = 96°
(ii) Oil contact angle (OCA) = 0°

## 3 Conclusion

Waste packaging polymeric EPE foam has been efficiently used for the removal of oil from different types of oil-water mixture with percentage removal up to 78%. The developed methodology utilizes waste polymers. So, we can minimize environmental pollution through simple approach. Furthermore, developed methodology can be efficiently used in large scale for separation of oils from contaminated water.
