# Supplementary material for: Waste packaging polymeric foam for oil-water separation: An environmental remediation
Source: Data Brief. 2018 May 18;19:86–92. doi: 10.1016/j.dib.2018.05.033 (PMC5993175; doi:10.1016/j.dib.2018.05.033)
Supplement: Supplementary file 1 — Supplementary material [file mmc1.doc]

**Covering Letter**

| **Article Title:** Waste packaging polymeric foam for oil-water separation: An environmental remediation |
| --- |
| **Name of the journal:** DATA IN BRIEF |
| **Abstract:** Nowadays, its urgent need to develop and fabricate efficient, low cost, eco-friendly, oil-water separation methodologies especially for variety of polluted water in the environments. To deals with serious oil spills and industrial organic pollutants, here in we have developed a highly efficient oil-water separation methodology by using waste material such as expanded polyethylene (EPE) polymeric foam which is most commonly used for packaging as a shock absorber and most abundantly available in the surroundings as waste. Oil-water separation setup was fabricated by using waste EPE polymeric foam without any pre-treatment. By simply scratching, special properties (wettability performance) such as hydrophobicity, leophilicity, and low water adhesion was imparted to the EPE polymeric foam. The different types of oil-water mixture used for the study and separation were achieved almost up to 78%. The oil absorption efficiency of the EPE polymeric foam was within range of 0.491-0.788 g/g. In addition to efficient oil-water separation, the modified EPE polymeric foam exhibited fast and continuous oil-water separation solely by gravity. The easy operation, chemical durability, and efficiency of the waste EPE polymeric foam give it high potential for use in industrial and consumer applications for large scale oil-water separation. |
| **Keywords:** Waste, packaging foam, oil-water separation, removal, wastewater, and environmental remediation. |
| **Name and Address of corresponding author:**  Dr. Anil H. Gore  ^1^Department of Chemistry, Rajashri Chhatrapati Shahu College, Kolhapur-416005, MS, India.  ^2^Fluorescence Spectroscopy Research Laboratory, Department of Chemistry, Shivaji University, Kolhapur-416004, MS, India.  Email: [anilanachem@gmail.com](mailto:anilanachem@gmail.com) (Dr. Anil H. Gore)  Contact No.+91-9975818177 |

I affirm that the manuscript has been prepared in accordance with International Journal of **DATA IN BRIEF** instructions to authors and the content of this manuscript, or a major portion thereof, has not been published in a referred journal no being submitted for publication elsewhere.


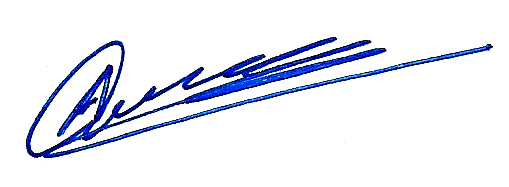


Signature of Corresponding Author Date

01/05/2018
